# Parity Attenuates Intraepithelial Corneal Sensory Nerve Loss in Female Mice

**DOI:** 10.3390/ijms21145172

**Published:** 2020-07-21

**Authors:** Mary Ann Stepp, Sonali Pal-Ghosh, Gauri Tadvalkar, Cintia S. de Paiva

**Affiliations:** 1 Department of Anatomy and Regenerative Biology, The George Washington University School of Medicine and Health Sciences, Washington, DC 20037, USA; spghosh@gwu.edu (S.P.-G.); grtdv@email.gwu.edu (G.T.); 2 Department of Ophthalmology, The George Washington University School of Medicine and Health Sciences, Washington, DC 20037, USA; 3 Ocular Surface Center, Department of Ophthalmology, Cullen Eye Institute, Baylor College of Medicine, Houston, TX 77030, USA; cintiadp@bcm.edu

**Keywords:** corneal sensory nerves, aging, pregnancy, mouse, parity, corneal sensitivity, corneal epithelial cell proliferation

## Abstract

Aging impacts the ocular surface and reduces intraepithelial corneal nerve (ICN) density in male and female mice. Many researchers use retired breeders to study naturally aged female mice. Yet, the impact of parity and the length of time since breeders were retired on age-related changes in the intraepithelial corneal nerves is not known. Here we study 2 month (M) nulliparous (NP) females as well as 9M, 10M, and 11M NP and multiparous (MP) female mice to determine whether parity impacts the age-related decline seen in corneal axon density; 9M male mice are also included in these assessments. After showing that parity attenuates age-related loss in axon density, we also assess the impact of parity on corneal epithelial cell proliferation and find that it impacts cell proliferation and axon density normalized by cell proliferation. Stromal nerve arborization is also impacted by aging with parity enhancing stromal nerves in older mice. qPCR was performed on 20 genes implicated in ICN density using corneal epithelial RNA isolated from 10M NP and MP mice and showed that NGF expression was significantly elevated in MP corneal epithelium. Corneal sensitivity was significantly higher in 9M MP mice compared to NP mice and increased sensitivity in MP mice was accompanied by increased nerve terminals in the apical and middle cell layers. Together, these data show that parity in mice attenuates several aspects of the age-related decline seen on the ocular surface by retaining sensory axons and corneal sensitivity as mice age.

## 1. Introduction

Pregnancy and lactation remodel female physiology and anatomy in numerous ways. Parity alters expression of genes that regulate hormones, neural gene expression, glial cell activation, immune function, and neurogenesis in the hippocampus [1,2]. Pregnant rodents produce elevated levels of corticosterones including cortisol which modifies stress and the immune response impacting metabolism and responses to inflammation [3,4]. To prevent fetuses from being rejected, the immune system is suppressed during pregnancy making dams more susceptible to inflammation. Cortisol is one of the hormones that suppresses immune function during pregnancy. Another hormone involved is prolactin (PRL), which is a proinflammatory hormone [5]; PRL levels are decreased in multiparous (MP) compared to primaparous or nulliparous (NP) rodents [6]. Estrogen, which has proinflammatory properties, is elevated in pregnant mice [7]. In addition, Nerve growth factor (NGF) plays a role in maintaining pregnancy [8] and induces neurogenesis in the hippocampus [1]. It also plays a role in the peripheral nervous system [9] and changes in its expression may impact the nerves that innervate the cornea.

Reports in 2003 [10] and 2009 [11] on the prevalence of dry eye disease in women and men, respectively, confirmed that the disease is higher in women (3.2 million in 2003) than men (1.7 million in 2009) and that, in women, each decade of life results in an increased risk of developing disease [10,12]. A cross-sectional, population-based study using the 2013 National Health and Wellness Survey involving 75,000 participants was carried out in 2017 [13] to determine the prevalence and risk of diagnosed DED in adults above the age of 18. That study showed that there were an estimated 6.8% or ~16.4 million people in the US suffering pathology associated with dry eye disease; 8.8% or ~11.1 million are women and 4.5% or ~5.3 million are men. While this study confirms that sex and older age matter, it also highlights that 2.7% or ~3.2 million people between the ages of 18–34 years have dry eye disease. Thus, dry eye disease is increasingly affecting the quality of life of working adults in the US and disproportionately impacting women [14].

Mouse models for the study of dry eye disease have led to improvements in treatment and understanding how the disease develops and progresses [15]. Using C57BL6J retired breeders (24M), we showed previously that naturally aged male and female mice lose corneal sensitivity and axon density of their intraepithelial corneal nerves (ICNs) [16]. ICNs consist of intraepithelial corneal basal nerves (ICBNs, previously referred to as subbasal nerves) and intraepithelial corneal nerve terminals (ICNTs) [17]. RNA isolated from corneal epithelial cells from 24M old female mice showed reduced expression of several neurotrophic factors [16]. The impact of the changes induced by pregnancy on the natural aging process in females has been a topic of study for decades [1,18,19,20]; to date, no one has looked at parity in female mice and its impact on the corneal sensory axons and corneal sensation. Here we use naturally aged NP and MP female C57BL6 mice from 2M–11M of age to show that progression of the age-related loss of axon density and corneal sensitivity is attenuated in MP mice.

## 2. Results

### 2.1. The Age-Related Loss of Axon Density Observed In Naturally Aged Female Nulliparous Mice Is Attenuated in Multiparous Mice

**Figure 1:**
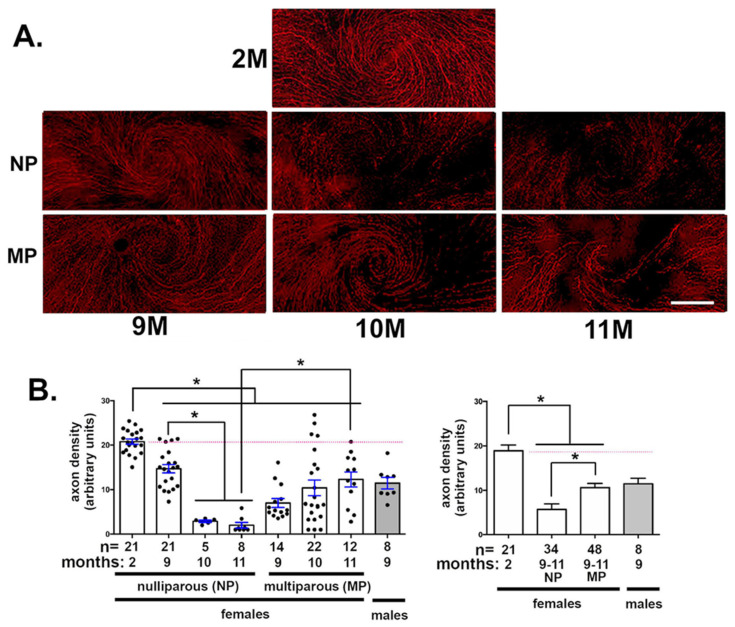
Axon density is higher in multiparous female mice compared to nulliparous female mice after 9 months (M) of age. Sholl analysis was performed on 2M nulliparous (NP) as well as 9, 10, and 11M NP and multiparous (MP) female mice and 9M male mice. (**A**) Representative Sholl images for female mice are shown. (**B**) Quantitation of axon density for each group is shown in B on the left. The numbers of mice are indicated. On the right, axon density data for clustered 9–11M NP and MP female mice are presented. Asterisks indicate significant differences with *p* values < 0.05. The dotted pink line highlights axon density for 2M NP controls. Bar in A = 175 μm.

MP and NP mice were sacrificed at 9M, 10M, or 11M after birth; 2M NP mice served as controls; 9M old male mice were also assessed. Representative images are shown in Figure 1A and quantification in Figure 1B. Axon density was assessed using Sholl analysis of en face whole mount confocal projection images as described in the methods section. Axon density in NP mice decreases with age. In both NP and MP females and male mice, axon density is decreased significantly at 9M compared to 2M NP females. By 10M and 11M, axon density in NP mice decreased further to levels that are approximately 10% of those in 2M NP mice. By contrast, there are no significant differences in axon density at 9M, 10M, and 11M compared to one another for MP mice; however, axon density in naturally aged MP mice is 30–45% lower at all time points compared to 2M in NP mice.

While axon density is reduced in both NP and MP mice at 9M, the difference is not significant. At 10M, axon density in MP mice varied dramatically; some corneas have axon densities similar to 2M NP mice, whereas others are similar to those in 10M NP mice. By 11M, the difference between MP and NP axon density is statistically significant: 11M MP corneas have higher axon densities than 11M NP corneas. Naturally aged male mice had corneal axon densities at 9M that are similar to those seen in 9M NP and MP mice and less than 2M NP female mice. Finally, when data are clustered so that 9–11M NP and 9–11M MP axon density values are combined, axon densities are less than those seen at 2M for both NP and MP mice. In addition, MP axon densities are significantly higher than those of NP mice.

Sholl analysis assesses both intraepithelial corneal basal nerves and nerve terminals. Because nerve terminals are largely perpendicular to the basal surface of the corneal epithelial basal nerves, they are underrepresented. Differences in the apical projection of nerve terminals could be present but require a different type of imaging and analysis for quantification.

### 2.2. Corneal Epithelial Cell Proliferation Changes Also Occur With Aging and Pregnancy

**Figure 2:**
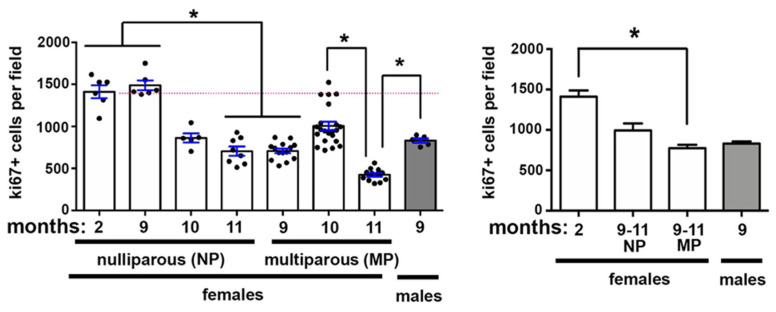
Corneal epithelial cell proliferation decreases after 9M as male and female mice age. The number of proliferating cells within the corneal epithelium was assessed by counting the mean number of ki67+ cells/field; 10 fields were assessed per cornea. On the left are data from each NP and MP age assessed. On the right, cell proliferation data are clustered for 9–11M NP and MP female mice. Asterisks indicate significant differences with *p* values < 0.05. The dotted pink lines highlight cell proliferation for the 2M NP controls.

MP and NP mice at 9M, 10M, or 11M after birth as well as 2M NP female and 9M old male mice were sacrificed. Cell proliferation was assessed by counting the number of ki67+ cell/field as described in the methods section. Data are presented in Figure 2. Cell proliferation in NP mice remains the same at 9M when compared to 2M; thereafter it decreases at 10M and 11M; the difference compared to 2M becomes significant at 11M. By contrast, in MP mice, cell proliferation is significantly reduced at 9M compared to 2M. While cell proliferation is higher at 10M, the increase is not significant compared to 9M or 2M. After 10M, cell proliferation decreases further and is significantly lower at 11M compared to 10M; 9M male mice also have lower proliferation rates compared to controls. Finally, when data are clustered so that 9–11M NP and 9–11M MP cell proliferation values are combined, cell proliferation is significantly reduced in MP mice compared to controls. While having fewer proliferating cells than 2M and more than 9–11M MP, the differences in proliferating cells in the 9–11M NP mice are not significant; 9M male mice have similar rates of cell proliferation as 9–11M NP and MP female mice.

### 2.3. The Impact of Axon Density on Corneal Epithelial Cell Proliferation Varies With Age and Parity

**Figure 3:**
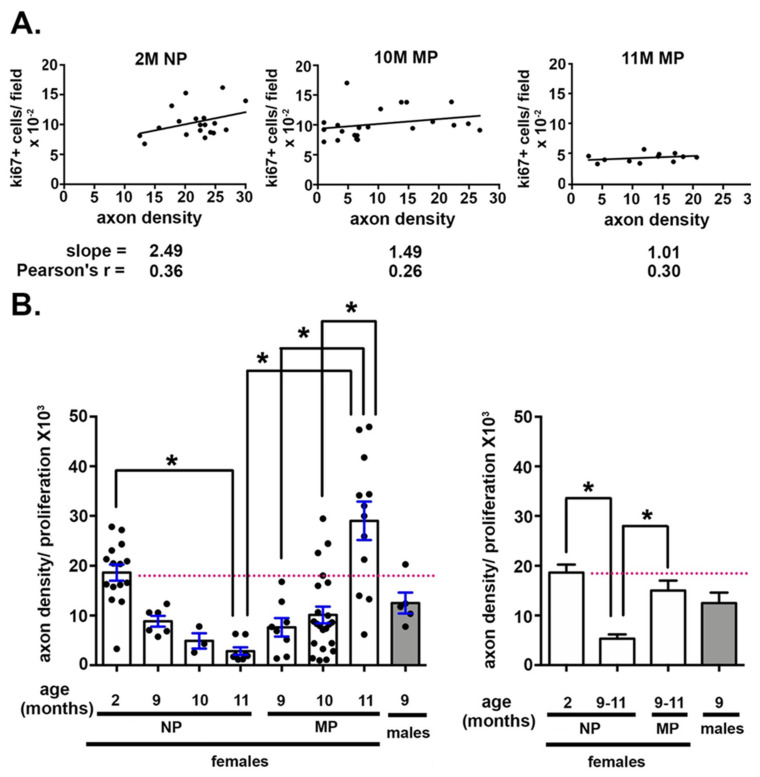
Parity both attenuates the age-related reduction in the ratio of axon density/corneal epithelial cell proliferation and increases the variation observed between individual corneas seen with aging. (**A**) The correlation between axon density and corneal epithelial cell proliferation was determined at 2M in NP and at 10M MP and 11M MP. While the slopes decreased at 10M and 11M compared to 2M, the Pearson’s r values were not significant. (**B**) For each cornea assessed, axon density was normalized by cell proliferation; data are presented on the left. On the right, normalized axon density data are clustered for 9–11M NP and MP female mice. Asterisks indicate significant differences with *p* values < 0.05. The dotted pink line highlights axon density for 2M NP controls.

To better understand the relationship between corneal epithelial basal cell proliferation and axon density, we plotted data for axon density vs cell proliferation for individual corneas. We had 22 cornea axon density values for the 10M MP mice and 12 from 11M MP mice. These values varied dramatically from a high of 27 to a low of 1. We exploited this variation to determine if corneas with higher axon densities had higher or lower rates of cell proliferation. Data are presented in Figure 3A for 2M NP, 10M MP, and 11M MP corneas. For these assessments, 10M and 11M NP data are not used because their axon densities are uniformly low, making it impossible to determine any correlation between cell proliferation and axon density.

While there was a trend for corneas with higher axon densities to also have higher rates of corneal epithelial cell proliferation, the Pearson correlation coefficients were 0.36 (2M), 0.26 (10M MP), and 0.30 (11M MP) indicating that the correlation between axon density and cell proliferation is not significant.

The slope for these data was 2.5 at 2M NP and at 10M MP it was 1.5. By 11M MP the slope was 1.01. The decrease in slope over time suggested to us that aging and/or parity were impacting how axon density and epithelial cell proliferation impact one another. We next normalized the axon density for each NP and MP cornea by dividing by its epithelial cell proliferation; the normalized axon density values drop over time for NP mice, whereas they rose over time for MP mice (Figure 3B). The normalized axon density values for MP mice at 9M, 10M, and 11M were not significantly different from those seen at 2M. By contrast, the normalized Sholl value for 11M NP is significantly lower than that seen at 2M. Thus, despite the fact that the correlation coefficients for the 2M NP, 10M MP and 11M MP mice indicate that axon density and cell proliferation are not correlated, parity impacts normalized axon density as mice age.

### 2.4. Stromal Nerve Arborization Is Reduced With Aging in NP and MP Mice 

**Figure 4:**
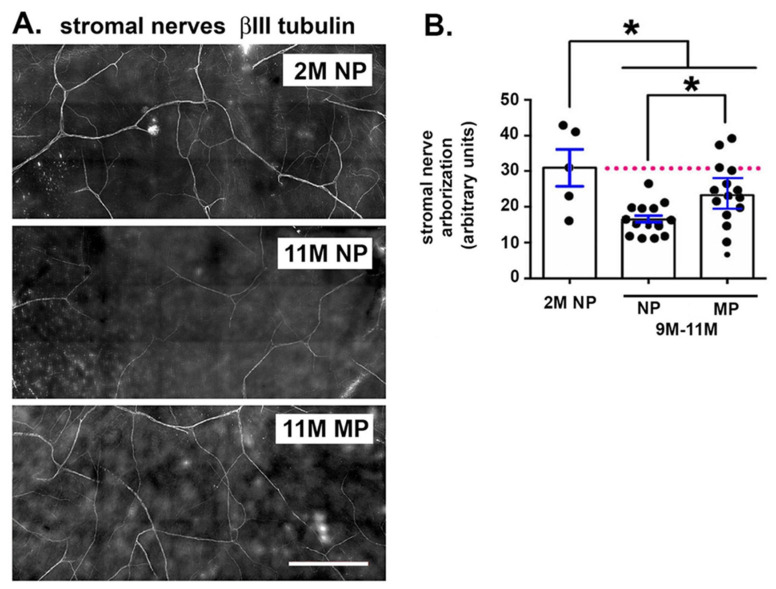
Age-related loss of stromal nerve arborization is partially attenuated in multiparous mice. Stromal nerve arborization was assessed at 2M in NP and at 9M, 10M, and 11M in NP and MP mice. Five corneas are assessed for each time point. (**A**) Representative stromal nerve images for 2M and 11M NP and 11M MP are presented. (**B**) Data for each variable are clustered for 9–11M NP and MP female mice and compared to data for 2M NP mice. Asterisks indicate significant differences with *p* values < 0.05. The dotted pink line highlights axon density for 2M NP controls. Bar = 500 μm.

Stromal nerve arborization was assessed in 5 corneas per variable for 2M NP and 9M, 10M, and 11M NP and MP mice; data are presented in Figure 4. There are fewer stromal nerves in both NP and MP corneas compared to 2M old mice. In addition, 9–11M NP mice have reduced stromal nerve arbors compared to 9–11M MP mice. 

### 2.5. The Attenuation of the Age-Related Loss of Axon Density Seen in Aged MP Mice Is Associated With Elevated Levels of Mrnas for Several Neurotrophic Factors

**Figure 5:**
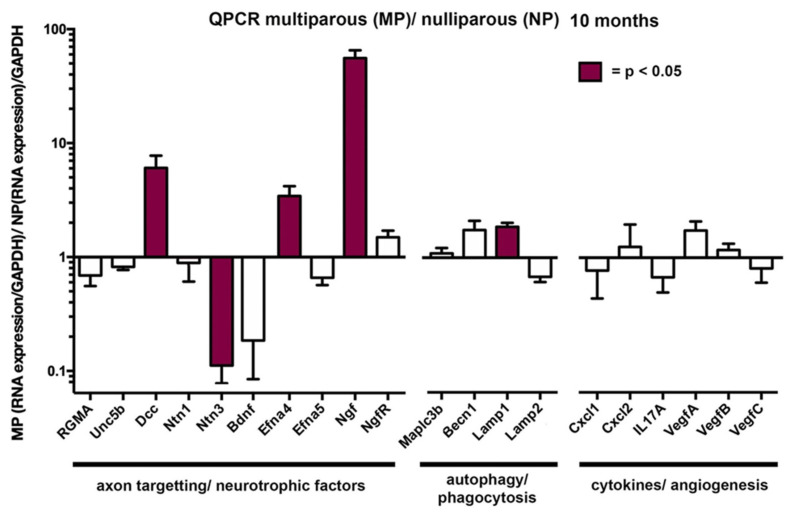
Parity leads to elevated expression of corneal epithelial mRNAs for Ngf, Dcc, Efna4, and Lamp1 and reduction expression of Ntn3 in female mice at 10M. RNA was isolated from the epithelium of 10M NP and MP mice and used for qPCR studies to look at expression of 20 RNAs involved in maintaining the corneal sensory nerves. Data for NP and MP RNA are normalized by GAPDH and presented as a ratio of MP/NP. Values above 1 indicate increased expression in MP and values less than 1 indicate reduced expression. Data that are significant are shown in maroon.

We next isolated RNA from 10M NP and MP mice and performed qPCR to quantify 20 different RNAs; these RNAs were chosen because of the roles they play in corneal sensory nerve homeostasis [16]; data are presented in Figure 5. For 15 RNAs, there was no significant difference in the expression of the RNA in MP compared to NP corneas. However, RNAs for four genes were significantly elevated in MP compared to NP mice including several neurotrophic factors: Deleted in colorectal cancer Dcc (6.1×), Ephrin A4 Efna4 (3.4×), and nerve growth factor Ngf (56×); by contrast, netrin 3 Ntn3 was significantly reduced in expression (0.11×) in MP corneal epithelium compared to NP. While brain derived neurotrophic factor (BDNF) was also reduced in expression (0.19×), the difference was not significant. One of the four RNAs assessed for autophagy, Lamp1, showed significantly elevated levels (1.9×) in MP mice, whereas the other three were not significantly different. None of the 6 mRNAs for cytokines and growth factors assessed were expressed at significantly different levels in RNA isolated from MP and NP corneal epithelium. These data implicate elevated expression of Ngf, Dcc, and Efna4 in maintaining ICNs in the MP mice.

### 2.6. Age-Related Loss of Corneal Sensitivity Is Attenuated in 9M MP Mice

**Figure 6:**
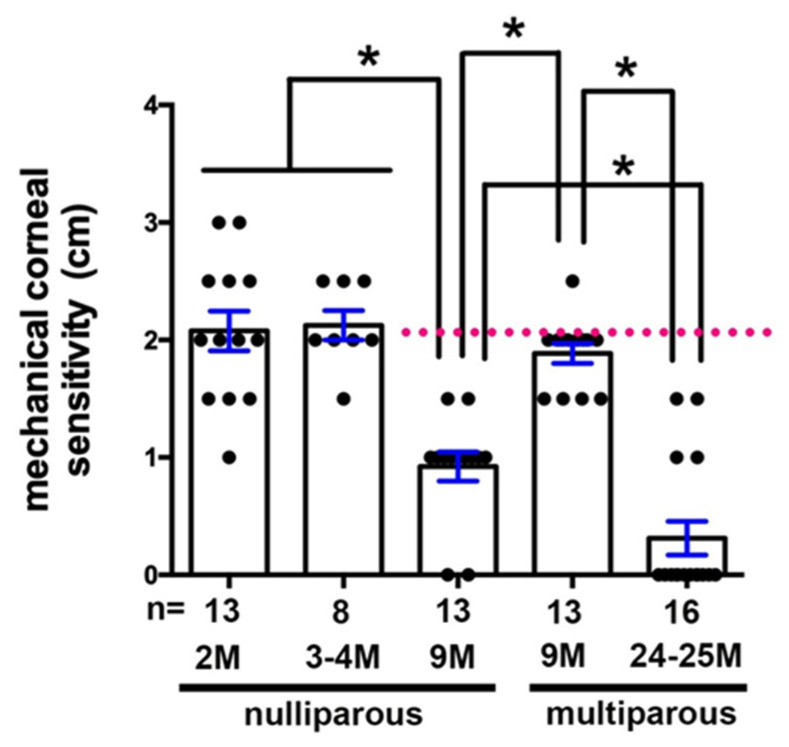
Parity attenuates age-related loss of cornea sensitivity in 9M but not 24M old female mice. Corneal sensitivity was assessed by modified Cochet–Bonnet anesthesiometer in 2M, 3–4M, and 9M NP and 9M and 24–25M MP mice. MP mice retain corneal sensitivity at 9M, whereas NP mice have significantly reduced sensitivity at 9M. Asterisks indicate significant differences with *p* values < 0.05.

We next assessed corneal sensitivity using a modified Cochet–Bonnet anesthesiometer in 2M, 3–4M, and 9M NP mice compared to 9M and 24–25M MP mice (Figure 6). Corneal sensitivity is maintained at 3–4M in NP mice but is reduced by 50% by 9M. In the MP mice at 9M, cornea sensitivity is reduced slightly but not significantly compared to 2M and 3–4M NP corneas. By 24M, corneal sensitivity is lower than at any other time point. While the 24M MP mice had been breeders, they were retired from breeding for several months prior to sacrifice.

### 2.7. Longer Parallel Intraepithelial Corneal Nerve Terminals (Picnts) Accompany the Enhanced Corneal Sensitivity at 9M in MP Mice

The data presented above in Figure 6 on 9M NP and MP mice shows significant differences in corneal sensitivity despite the fact that the data presented in Figure 1 on axon density show a reduced but not significant reduction in MP compared to NP mice. Sholl analysis allows assessment of the intraepithelial corneal basal nerves (ICBNs) but the projection images mask differences in the intraepithelial corneal nerve terminals (ICNTs), which extend upwards to the ocular surface. Once the nerves reach the apical squames, their growth is halted by tight junctions and the ICNTs turn 90 degrees and begin to extend parallel to the corneal epithelial surface beneath the apical squames. We refer to these nerve terminals as parallel intraepithelial corneal nerve terminals (pICNTs). The pICNTs can be imaged using confocal imaging and 3D image analysis.

**Figure 7:**
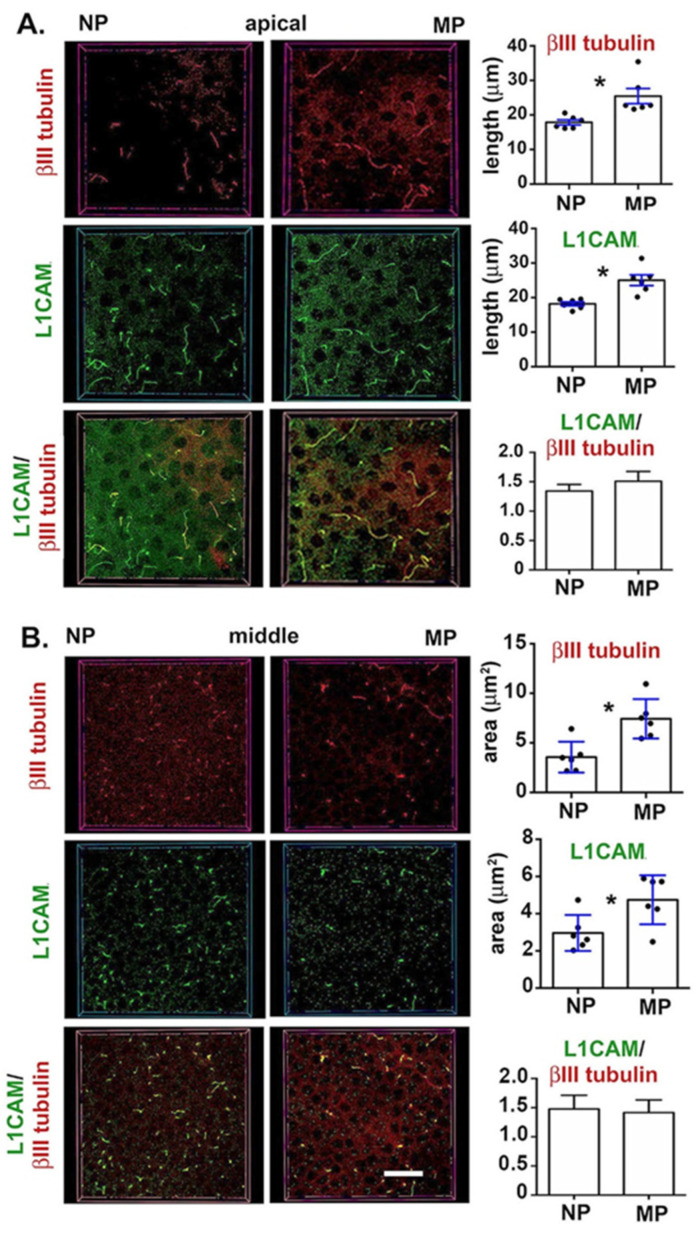
Parallel intraepithelial corneal nerve terminals (pICNTs) are longer in 9M in MP mice. After staining corneas with antibodies against both βIII tubulin and L1CAM, the lengths of the pICNTs and ICNT areas were assessed in en face confocal images at apical (**A**) and middle (**B**) regions of the cornea, respectively. pICNTs and ICNTs are longer and larger in area in MP compared to NP mice. Bar = 50 μm. Asterisks indicate significant differences with *p* values < 0.05.

In Figure 7, we show representative 3D confocal images taken from the center of the cornea at the vortex from six 9M NP and MP corneas, respectively, whose nerves were visualized using βIII tubulin and L1CAM. Individual confocal images showing the apical aspect of each cornea are presented on the left and from the middle of the cornea are presented on the right. For the apical images, we measure the lengths of the pICNTs using both βIII tubulin and L1CAM; for the middle images, nerve terminals appear as puncta. We measured the sizes of the puncta using both βIII tubulin and L1CAM. Because we have shown data on the ICBNs in Figure 1, we do not present those data again. While βIII tubulin is an intracellular cytoskeletal protein, L1CAM is an integral membrane cell adhesion molecule whose extracellular domain interacts homophilically with L1CAM or heterophilically with integrins on corneal epithelial cells. The epitope recognized by the antibody used for L1CAM is extracellular and the protein is susceptible to cleavage from ICNs by extracellular proteases during wound healing on reinnervating ICNs [21]. We then calculate the ratio of L1CAM/βIII tubulin in the apical and middle regions of the cornea for six corneas each for NP and MP mice.

Data show that the pICNTs are significantly longer in the apical cell layers and significantly larger in middle layers of the MP corneas compared to NP at 9M whether assessed by βIII tubulin or L1CAM. These data indicate that nerve terminals are larger and grow longer in the MP corneas. The ratios of L1CAM/βII tubulin in the apical and middle regions are similar for 9M NP and MP 9M corneas. The reduced sizes of the ICNTs and shorter lengths of the pICNT in NP corneas do not appear to be due to increased protease activity in the corneal epithelium since L1CAM is retained similarly on βIII tubulin+ MP and NP nerve terminals.

## 3. Discussion

The data presented here show that parity in female mice delays age-related loss of axon density and corneal sensitivity and it does so via a mechanism that appears to involve elevated NGF levels. NGF was the first growth factor characterized [22,23] and has been studied as a trophic factor for the cornea [24,25,26]. NGF can induce axonal elongation in both the central and peripheral nervous systems [23]. Recent clinical trials using a recombinant form of NGF have shown promise in treating dry eye disease [27] and neurotrophic keratitis [28]. RNAseq studies show that NGF mRNA is produced by corneal epithelial and stromal cells and is increased in expression within 18 h after simple debridement injury in mice [21]. At 10M and 11M, there is a large amount of variation in axon density values in MP mice with some as high as those seen at 2M but many values as low as those seen in 10M and 11M NP mice. Despite the fact that the 10M MP corneal epithelium expresses 50-fold more Ngf RNA than 10M NP corneal epithelium, many of the MP corneas undergo significant axon loss. While some studies say that NGF levels in serum do not vary with pregnancy and others show slight increases [29], the magnitude of the changes seen in the corneal epithelium in the MP mice indicate that the increase in Ngf mRNA is secondary to changes in other pregnancy hormones.

One candidate is cortisol, which is elevated during pregnancy [3] and has been shown to induce expression of NGF in humans [30] and rodents [31]. In addition to supporting neurite extension and growth, NGF, along with BDNF, regulate immune function as part of what is referred to as the neuro-endocrine-immune axis. It is this axis that mediates the physiological changes that take place during pregnancy and lactation and is implicated in the increased risk women have in acquiring autoimmune diseases. Research has shown that reduced function of several different classes of immune cells and loss of circadian rhythm occur with aging [32,33]. The qPCR data presented here shows no change in the expression of several cytokine RNAs in 10M MP compared to 10M NP mice. This suggests that alterations in resident immune cells do not play a role in the retention of ICNs and corneal sensitivity in the MP mice. The ICNs themselves are known to play trophic roles for the corneal epithelial cells by releasing neuropeptides and growth factors that can be used as signaling molecules and energy sources for the corneal epithelial cells regulating their proliferation [34]. With aging, corneal epithelial cells lose their dependence on ICNs for trophic factors and upregulate the RNA for Ngf.

We previously showed that Ntn1, Efna5 and Dcc are reduced in 24M female C57BL6 mice compared to controls and the reduction is correlated with axon density loss [16]. Dcc and Unc5b are dependence receptors [35,36]; when netrins, which are laminin homologs, bind to Dcc and Unc5b, axon growth and branching are favored; when receptors are not occupied, they can induce apoptosis. Here we show that MP mice have similar expression of Unc5b and elevated expression of Dcc and Efna4 in addition to elevated Ngf compared to NP mice. While we assessed netrin 1 and 3 RNAs, we did not assess netrin 4, netrin G1, or netrin G2 which are also expressed by corneal epithelial and stroma cells [21]. Ephrins, including Efna4, function to potentiate axon guidance mediated by netrins [37,38,39]. Elevated expression of these neurotrophic factors in 10M MP mice is consistent with their increased sensitivity and axon density in aging MP mice compared to NP mice.

Since cell proliferation rates in 9–11M MP mice are significantly lower than those in 9–11M NP mice, elevated expression of NGF within the corneal epithelium of MP mice is likely not serving trophic functions for the corneal epithelial cells. It may be functioning to maintain growth of the ICNs. The terminal ends of the ICNTs in young 2M old male and female mice are shed daily in a mechanism that is under diurnal control [40]. Once shed, the ICNTS begin to regrow. This growth is supported by expression of neurotrophic factors by the corneal epithelial cells and is lost in older mice [16]. Circadian rhythms are reduced with aging and contribute to reduced reproductive ability in rodents; pregnancy itself alters the timing of the circadian clock in mice [41]. The age-related decline in reproductive ability in mice can be restored by manipulating the light–dark cycle to match the changes occurring as the mice age [42]. It is not clear whether older mice lose circadian control of ICNT shedding and regrowth but the data shown here on the 10M and 11M NP female mice suggest that this is taking place and that the enhanced neuro-endocrine-immune functions induced by pregnancy slow the rate of loss of circadian control within the cornea.

The impact of reproduction on life expectancy has been studied for many years and a number of hypotheses have been put forth to explain why multiple pregnancies do not reduce life span in mammals [43,44]. These hypotheses often refer to pregnancy as inducing oxidative stress and oxidative shielding to protect the mother from the harmful effects of pregnancy. Increased oxidative damage to cells and tissues are observed during and after pregnancy in a variety of mammals and birds. One meta-analysis of the literature on this topic looked at the oxidative state of tissues and markers in non-breeding females compared to females that had just started breeding and found support for the hypothesis. The transition to the reproductive state in birds and mammals was associated with what authors refer to as a “step-change reduction” in oxidative damage in certain tissues and markers [45,46] Oxidative shielding has been proposed to protect females from the hazards associated with increased metabolism and oxidation that takes place during pregnancy and lactation [44,45,47,48] The accumulation of chronic oxidative damage has long been thought to lead to many of the changes seen in aging. The mechanisms that permit oxidative shielding use the innate immune system [45].

Dry eye disease is a chronic disease that affects corneal nerves [16,49,50]. Clinically, dry eye patients complain of eye irritation and blurred vision; these symptoms vary from insignificant to severe. The pathophysiology of dry eye is not entirely understood. However, it has become evident that both innate and adaptive immunity immune responses are involved [51]. Animal models have shown active participation of CD4+ T cells [52,53]. Although both sexes are affected, dry eye affects more women than men, and its prevalence increases sharply in women in the 5th decade [10,11,13,54,55,56] Because of its strong association in women and perimenopause, the influences of sex hormones have been postulated as playing roles in either promoting or ameliorating dry eye disease [57,58,59]. Our results evaluating aging MP and NP mice suggest that hormonal changes associated with pregnancy may influence the overall health of corneal nerves. Pregnancy affects the course of several autoimmune diseases. For example, reports show that rheumatoid arthritis, multiple sclerosis, Grave's disease, and Hashimoto thyroiditis improve during pregnancy only to flare in the postpartum period; yet, Systemic Lupus Erythematosus and Systemic Sclerosis worsen during pregnancy [60]. The specific effects of pregnancy on dry eye severity and its prevalence in humans have not been evaluated. Our results indicate that further investigation is warranted. Improved understanding of the impact of sex hormones on age-related pathologies in the cornea is needed.

## 4. Materials and Methods 

Animals: All studies performed comply with the George Washington University (IACUC protocol number A252, valid until 05/12/2022) and Baylor College of Medicine (IACUC protocol number AN-7342, valid from 1/11/17 until 01/27/2023) Institutional Animal Care and Use Committee guidelines and with the ARVO Statement for the Use of Animals in Vision Research. C57BL6 mice were aged and bred in pathogen free environments for up to 11Months of age.

Immunofluorescence: All eyes were fixed immediately after enucleation in a paraformaldehyde-containing fixative (1× PBS, 1% formaldehyde, 2-mM MgCl2, 5-mM EGTA, 0.02% NP-40) for 1 h and 15 min at 4 °C, followed by two washes for 10 min each in 1× PBS containing 0.02% NP40 at room temperature. Tissues were then placed in 4:1 methanol:dimethyl sulfoxide (DMSO) for 2 h at −20 °C and then stored in 100% methanol at −20 °C until used for whole-mount staining studies. The back of the eye was cut and the retina, lens, and iris removed before staining. Tissues were transferred to a graded Methanol-TritonX-100 series (75%, 50%, and 25% methanol:TritonX-100 for 15, 15, and 10 min, respectively). All incubations were performed with gentle shaking and at room temperature, unless otherwise specified. The eyes were washed twice in PBS, for 30 min each, followed by incubation with blocking buffer for 2 h. Blocking buffer was made as follows: To 100 mL 1× PBS, 1 g of BSA was added, the mixture was stirred for 10 min, 1 mL of horse serum was added, and the mixture was stirred for an additional minute.

The tissues were then incubated overnight with primary antibody diluted in blocking buffer at 4 °C. The following antibodies were used: βIII tubulin (TUJ1; #801201; Biolegend, San Diego, CA, USA), L1CAM (#MAB5272; Millipore, Temecula, CA, USA) and ki67 (#ab16667; Abcam, Cambridge, MA, USA). Appropriate secondary DyLite 488, 594, and 647 antibodies from Jackson Immunobiologicals (West Grove, PA, USA) were used for immunolabeling. The next day, the tissues were washed five times with PBS and 0.02% Tween 20 (PBST) for 1 h each, blocked for 2 h, and then incubated with secondary antibody diluted in blocking buffer overnight at 4 °C. The following day, eyes were washed three time with PBST for 1 h each, followed by nuclear staining with 4,6-diamidino-2- phenylindole (DAPI) for 5 min, and washed with distilled water. To achieve the best flattening, the corneas were placed epithelial side-up with Fluoromount G mounting media (#17984-25; Electron Microscopy Sciences, Hatfield, PA, USA) and coverslipped.

Confocal microscopy: Confocal microscopy was performed at the GW Nanofabrication and Imaging Center at The George Washington University Medical Center. A confocal laser-scanning microscope (Zeiss 710; Carl Zeiss Inc. San Diego, CA, USA) was used to image the localization of Alexa Fluor 488 (Jackson Immunobiologicals; argon laser; 488-nm laser line excitation; 495/562 emission filter;), and Alexa Fluor 594 (Jackson Immunobiologicals; 561 diode laser; 594-nm nm laser line excitation; 601/649 emission filter) and Alexa Fluor 647 (Jackson Immunobiologicals; 633 Diode laser; 647-nm laser line excitation; 671/759 emission filter). Optical sections (z = 0.5 μm) were acquired sequentially with a 63× objective lens. Three-dimensional (3D) images were generated using Volocity software (Version 6.3; Perkin Elmer, New York, NY, USA). High-resolution images were presented as en face or as cross sections projected through the length of the acquired image (135 μm), or as cross-sections projected through 0.5 μm of tissue. Image J was used for the measurement of the lengths of nerve terminals and Neuron J for the stromal nerve quantitation. Each image subjected to quantification was obtained using the same confocal laser settings and the same intensity settings in Volocity to permit valid comparisons. Sum intensity for each color for basal en face images was obtained using ROI statistics in the Nikon Analysis Software (NIS-Elements AR Analysis 5.20.01)

For Sholl analysis, images were acquired using the Zeiss Cell Observer Z1 spinning disk confocal microscope (Carl Zeiss Inc. Thornwood, NY, USA), equipped with ASI MS-2000 (Applied Scientific Instrumentation, Eugene, OR, USA) scanning stage with z-galvo motor, and Yokogawa CSU-X1 spinning disk. A multi-immersion 25×/0.8 objective lens, LCI Plan-Neofluor, was used for imaging, with oil immersion. Evolve Delta (Photometrics, Tucson, AZ, USA) 512 × 512 EM-CCD camera was used as detector (80-msec exposure time). A diode laser emitting at 568 nm was used for excitation (54% power). Zen Blue software (Carl Zeiss Inc.) was used to acquire the images, fuse the adjacent tiles, and produce maximum intensity projections. The adjacent image tiles were captured with overlap to ensure proper tiling. Sholl analysis was performed using ImageJ as described previously. Images at acquired at sites where the vortex is present.

Cell Proliferation: For cell proliferation studies, images were acquired on the Nikon E600 Fluorescent Microscope and the numbers of ki67+ cells per field were analyzed using ImageJ. Two fields were imaged in each peripheral zone (4 zones) and 2 images at the corneal center, so 10 fields total per eye were assessed.

Stromal Nerve Arborization: For measuring the length of the stromal nerves in Figure 4, we did the following: Loaded Neuron J (through Image J), opened Image, converted to 8 bit and saved as jpg, used ‘Add tracing’ tool from the tool bar to draw along the length of the nerve, double clicked to end the tracing, and used ‘Measure tracing tool’ to determine the length of the nerve in μm.

RNA isolation: Total RNA from cornea epithelial cells obtained by debridement (both eyes pooled) was extracted using a QIAGEN RNeasy Plus Micro RNA isolation kit (Qiagen) following the manufacturer’s protocol. Epithelium from two 10M NP and two 10M MP mice per age were pooled for each replicate sample and a minimum of 5 replicates were used for NP and MP corneas. After isolation, the concentration of RNA was measured a NanoDrop^®^ ND-2000 Spectrophotometer (Thermo Scientific, Wilmington, DE, USA) and stored at −80 °C until used.

qPCR: For quantitative polymerase chain reaction (qPCR) studies, at least 5 samples per time point were used. qPCR was performed using a Bio-Rad CFX384 Real-Time PCR detection system. The following primers used were obtained from BioRad (Hercules, CA, USA): Bec1(qMmuCID0005981), LC3A(Map1Lc3a; qMmuCED0045817), Lamp1 (qMmuCID0027030), Lamp2(qMmuCID0011408), Cxcl1(qMmuCED0047655), Bdnf(qMmuCED0050333), Gapdh (qMmuCED0027497). Additional primers were obtained from Qiagen (Germantown, MD, USA): Ntn1, #QT00128478), Dcc (#QT00135100), Unc5b (#QT00167846), Efna4(#QT00100681), Efna5 (#QT00116494), Rgma (#QT00310583), LC3B(Maplc3b; #QT00055069), Ngf (#QT00093464), Ngfr (#QT01047004), Rgma (#QT00310583), VegfA (#QT00160769), VegfB (#QT01059863), VegfC (Q#QT00104027), Cxcl1 (#QT00093436), IL17a (#QT00103278). QPCR data is normalized against Gapdh.

Corneal mechanical sensitivity: Corneal sensitivity was measured under a surgical loupe with a 9-0 nylon monofilament of different lengths (1.0, 1.5, 2.0, 2.5, 3.0, 4.0 cm). While holding the animal, a nylon filament was applied to the cornea and a positive response was indicated by a clear stimulus-evoked blink and retraction of the eye into the ocular orbit. The central cornea was tested six times at each filament length. The response was considered negative when no blink was elicited by the monofilament touch. A positive response was considered when the animal blinked more than or equal to 50% the number of times tested. If no blink response could be elicited at a monofilament length of 1.0 cm, corneal sensitivity was recorded as 0.

Statistical analyses: Quantitative data are presented as mean ± standard error of the mean. All data were analyzed using one-way ANOVA. All statistical tests were performed using the GraphPad Prism Program, Version 6 (GraphPad Software Inc. San Diego, CA, USA). A *p* value < 0.05 was considered statistically significant.

## Abbreviations

ICNs	intraepithelial corneal nerves
ICBNs	intraepithelial corneal basal nerves
ICNTs	intraepithelial corneal nerve terminals
NP	nulliparous (virgin female)
MP	multiparous (breeding female)
PRL	prolactin
NGF	nerve growth factor
DMSO	dimethyl sulfoxide
PBS	phosphate buffered saline
M	months
DAPI	4,6-diamidino-2- phenylindole
qPCR	quantitative polymerase chain reaction
